# Assessing coastline recession for adaptation planning: sea level rise versus storm erosion

**DOI:** 10.1038/s41598-023-35523-8

**Published:** 2023-05-22

**Authors:** Roshanka Ranasinghe, David P. Callaghan, Fan Li, David J. Wainwright, Trang Minh Duong

**Affiliations:** 1 Department of Coastal and Urban Risk & Resilience, IHE Delft Institute for Water Education, P.O. Box 3015, 2601 DA Delft, The Netherlands; 2 Resilient Ports and Coasts, P.O. Box 177, 2600 MH Delft, The Netherlands; 3 Department of Infrastructure Engineering, University of Melbourne, Melbourne, Australia; 4 School of Civil Engineering, The University of Queensland, Brisbane, QLD 4072 Australia; 5 College of Hydraulic Science and Engineering, Yangzhou University, Yangzhou, China; 6 Salients Pty Ltd, P.O. Box 566, Wallsend, NSW 2287 Australia; 7 Water Engineering and Management, University of Twente, P.O. Box 217, 7500 AE Enschede, The Netherlands

**Keywords:** Natural hazards, Engineering

## Abstract

The Sixth Assessment report (AR6) of the Intergovernmental Panel on Climate Change (IPCC) states with high confidence that most sandy coasts around the world will experience an increase in coastal erosion over the twenty-first century. An increase in long term coastal erosion (coastline recession) along sandy coasts can translate into massive socio-economic impacts, unless appropriate adaptation measures are implemented in the next few decades. To adequately inform adaptation measures, it is necessary to have a good understanding of the relative importance of the physical processes driving coastline recession, as well as of linkages between consideration (or not) of certain processes and the level of risk tolerance; understandings that are hitherto lacking. Here, we apply the multi-scale Probabilistic Coastline Recession (PCR) model to two end-member sandy coastal types (swell dominated and storm dominated), to investigate where and when coastline recession projections are dominated by the differential contributions from Sea Level Rise (SLR) and storm erosion. Results show that SLR substantially increases the projected end-century recession at both types of coasts and that projected changes in the wave climate have only a marginal impact. An analysis of the Process Dominance Ratio (PDR), introduced here, shows that the dominance of storm erosion over SLR (and vice versa) on total recession by 2100 depends on both the type of the beach and the risk tolerance levels. For moderately risk-averse decisions (i.e. decisions accounting only for high exceedance probability recessions and hence do not account for very high amounts of potential recession—for example, the placement of temporary summer beach cabins), additional erosion due to SLR can be considered as the dominant driver of end-century recession at both types of beaches. However, for more risk-averse decisions that would typically account for higher potential recession (i.e. lower exceedance probability recessions), such as the placement of coastal infrastructure, multi-storey apartment buildings etc., storm erosion becomes the dominant process. The results of this study provide new insights on which physical processes need to be considered when and where in terms of numerical modelling efforts needed for supporting different management decisions, potentially enabling more streamlined and comprehensive assessments of the efficacy of coastal adaptation measures.

## Introduction

The low-elevation coastal zone (LECZ, i.e. below 10 m from MSL) of the world is currently home to over 10% of the global population^1,2^; an estimate that is projected to increase to 1 billion by 2060^2^. Correspondingly, the consequences of coastal erosion will also increase in the decades ahead, especially along heavily inhabited and utilized (e.g. tourism, fishing, ports and harbours, dwelling) sandy coasts. Climate change will exacerbate such consequences further due to projected increases in long term coastal erosion (coastline recession) over the twenty-first century along sandy coasts in a vast majority of the world’s regions^3^. For example, in a first-pass global scale assessment, Vousdoukas et al.^4^ have projected that, under the high emissions RCP 8.5 scenario, up to 1/3rd of the sandy beaches in LECZs (current population density of ~ 500 people/km^2^) could face severe erosion (defined therein as a shoreline retreat exceeding 100 m relative to the present-day shoreline position) by mid-century, increasing to about 2/3rds by the turn of the twenty-first century. In terms of economic consequences of coastline recession, Hinkel et al.^5^ have estimated that the cost of migration over the twenty-first century due to coastal erosion could reach US $ 1 Trillion. To avoid or minimize socio-economic impacts that are certain to arise from coastline recession^5–8^, especially in heavily inhabited and utilized locations, adaptation strategies and measures that mitigate coastal risks are needed, particularly considering the lucrative economic rewards often provided by investments in the coastal zone. Furthermore, as adaptation planning needs to account for the fairly long lead-times associated with strategies such as managed retreat^9^, action is needed sooner rather than later.

While there is now broad awareness that climate change is very likely to result in coastline recession along open sandy coasts (in the absence of additional sources of sand supply or physical barriers to recession)^4,10–13^, it is still more or less generally presumed that this recession will be driven by sea level rise. But this simplistic view does not hold in all situations. This is because, in addition to SLR, there is also a hysteresis effect of the storm erosion-beach recovery cycle that contributes to the long term position of the coastline, and hence to the amount of coastline recession^14–16^. Could this storm erosion leave a larger signal on total coastline recession at some types of coasts? Or could the effect of storms be largely ignored (thus greatly reducing the complexity of the modelling effort required) at some coastal types? Does the inclusion of storm erosion in computing coastline recession become more important for increasingly risk averse decision making? These are all questions that become very relevant where decision making at local scale (i.e. at a given site) is concerned; questions that to date remain unanswered. This study attempts to answer some of these questions, through the application of the Probabilistic Coastline Recession (PCR) model, under the high-emission SSP5-8.5 scenario over the twenty-first century, at two distinctly different study sites representing two main categories of sandy coastal types around the world.

The PCR model, fully described in Ranasinghe et al.^14^, is a physics-based, multi-scale, probabilistic model that provides probabilistic projections of coastline position change (generally over a 100 year period) due to the combined effect of SLR and storm erosion. The model is multi-scale in that it concurrently and seamlessly accounts for coastline position change due to both SLR and storm erosion (due to the combined effects of storm surge and storm waves), while allowing beach recovery between storm events. The PCR model uses data-fitted distributions of storm wave conditions and water levels, to generate a 100-year synthetic storm time series that is then superimposed on a chosen SLR trajectory to calculate the associated movements of the coastline over ~ 100 years within a Monte Carlo framework. The ~ 100-year simulation is repeated multiple times (~ 1000) until convergence is obtained for the projected coastline recession at low (e.g. 0.05) exceedance probabilities (see “Methods” for a more detailed description of the model). The model, and its derivatives, have been applied at several locations around the world (Australia, France, Japan, Sri Lanka)^15–21^.

Here we apply the PCR model to two beaches that are representative of two main categories of open coasts (in terms of hydrodynamic forcing) present around the world: swell dominated (Narrabeen beach, Australia) and storm dominated (Noordwijk aan Zee strand, The Netherlands) (see “Methods” for study site descriptions). In terms of governing morphodynamic processes too these two beaches are diametrically opposite with Narrabeen being swash dominated and Noordwijk aan Zee being drift dominated, albeit without any noteworthy alongshore gradient in longshore sediment transport rate, for the most part. The model implementation for the two sites is identical to that described in Ranasinghe et al.^14^ and Wainwright et al.^15^ (for Narrabeen, Profile #4) and Li et al.^17^ (for Noordwijk aan Zee, JARKUS profile # 8250). In both cases, the toe of the dune is taken as the coastline position indicator, and the model is run for the entirety of the twenty-first century with 3 different wave climates, with and without SLR (SSP5-8.5), as summarized in Table 1. Note that, as indicated in Table 1, here we only changed the storm wave heights to account for climate change driven variations in storms, while all other storm parameters (e.g. wave period, direction, post-storm profile recovery) were kept unchanged at present-day values. As storm surge projections for both sites indicate negligible changes over the twenty-first century^22,23^, the present-day storm surge climate was assumed to remain unchanged over the twenty-first century.

**Table 1:** Summary of the PCR simulations undertaken in this study.

	Waves	SLR (by end of twenty-first century, relative to end of twentieth century)
Simulation #1: Present-day storms only	Present-day wave climate	None
Simulation #2: Present-day storms and SLR	Present-day wave climate	SSP5-8.5, higher bound of likely range (IPCC AR6) (~ 1 m)
Simulation #3: Future storms (higher) and SLR	Increased peak storm H_s_ (by 3% for Narrabeen^a^; by 5% for Noordwijk^b^)	SSP5-8.5, higher bound of likely range (IPCC AR6) (~ 1 m)
Simulation #4: Future storms (lower) and SLR	Decreased peak storm H_s_ (by 13% for Narrabeen^a^; by 5% for Noordwijk^b^)	SSP5-8.5, higher bound of likely range (IPCC AR6) (~ 1 m)

^a^Hemer et al.^24^, ^b^Debernard and Roed^25^.

## Results

**Figure 1:**
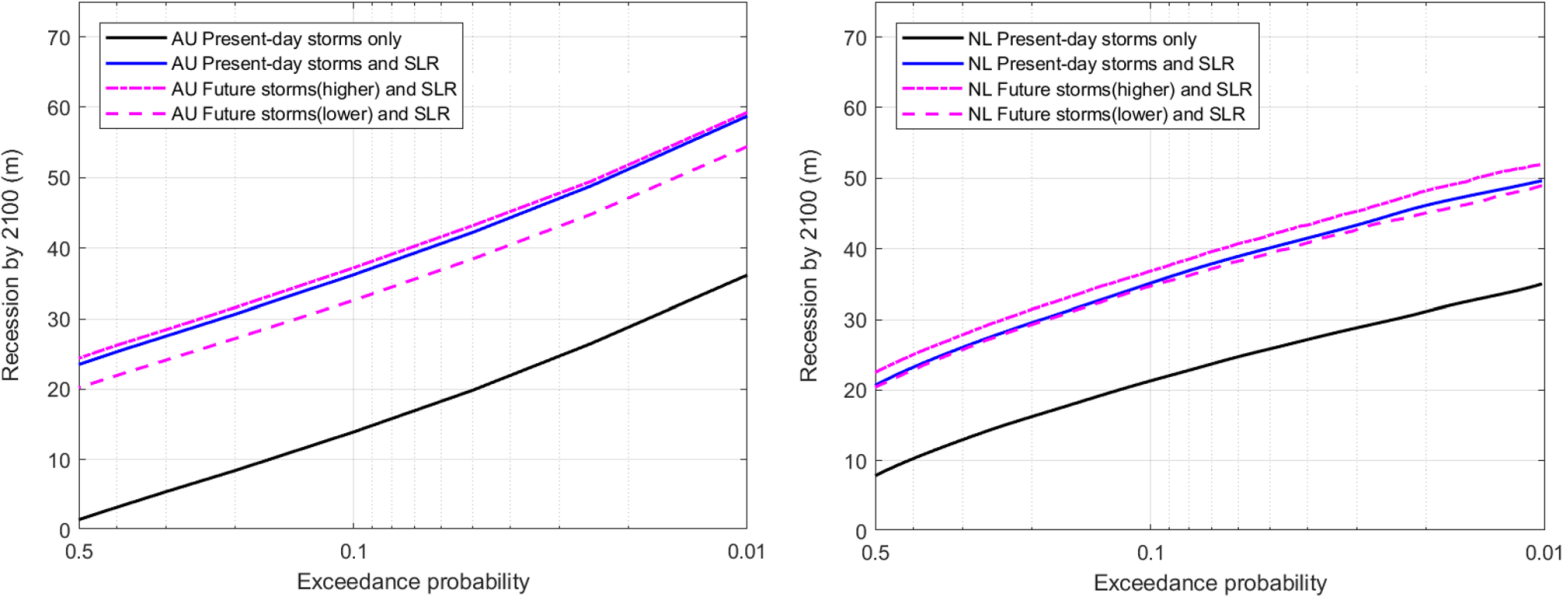
Exceedance probability of projected end-century coastline recession due to sea level rise (upper end of the IPCC AR6 likely range for SSP5-8.5) and present-day/future storm erosion at: (left) Narrabeen beach, Sydney, Australia (annotated AU); (right) Noordwijk aan Zee strand, The Netherlands (annotated NL).

The projected coastline recessions by the end of the twenty-first century (relative to end of the twentieth century) for the two sites under different forcing conditions are shown in terms of exceedance probabilities ranging from 0.01 to 0.5 (expected value) in Fig. 1. Comparison of Simulations #1 and #2 immediately show that SLR substantially increases the projected end-century recession at both sites (by about 25 m and 15 m at Narrabeen and Noordwijk aan Zee respectively). However, the consideration of projected changes in the wave climate results in rather small differences in the recession projected by the end of the century at both sites (Simulations #2, #3 and #4). In general, at exceedance probabilities of around 0.5, the maximum recession projected by end-century at both sites are low (about 20–25 m), increasing to 50–60 m at low exceedance probabilities of around 0.01. PCR projected coastline recessions at these two sites have been previously compared with Bruun rule estimates^14,17^ and show that in both cases the Bruun rule estimates lie at the lower end of PCR modelled exceedance probabilities (i.e. the model predicted median values are much lower than what the Bruun rule predicts).

Note that the difference in the shapes of the recession distributions (i.e. shape of the curves in Fig. 1 left vs right) for the two study locations is due to the semi-log axis plotting done here, and when the curves are plotted on a linear scale the shapes of the curves are in fact similar. However, a noteworthy difference in the response characteristics of the two sites is that the rate of recession (i.e. first derivative) slowly increases for lower exceedance probabilities at Narrabeen, as indicated by the slightly concave shape of the curve from about 0.1 to 0.01 probability (Fig. 1 left), while at Noordwijk aan Zee, the rate of recession slowly decreases in the same low probability range as indicated by the slightly convex shape of the curve (Fig. 1 right). This is because the storm wave height hardly increases for lower exceedance probabilities at Noordwijk aan Zee^26^ while at Narrabeen they keep increasing for the full exceedance probability range considered here^29^.

The contribution of SLR to the total recession by end of the century can be approximated by subtracting the results of the simulation with storms only (Simulation #1) from that of the simulations that account for both storms and SLR (Simulations #2 to #4). The SLR-alone recession computed in this way would include the effect of non-linear interactions between SLR and storms, which is in fact a unique feature of the PCR model.

**Figure 2:**
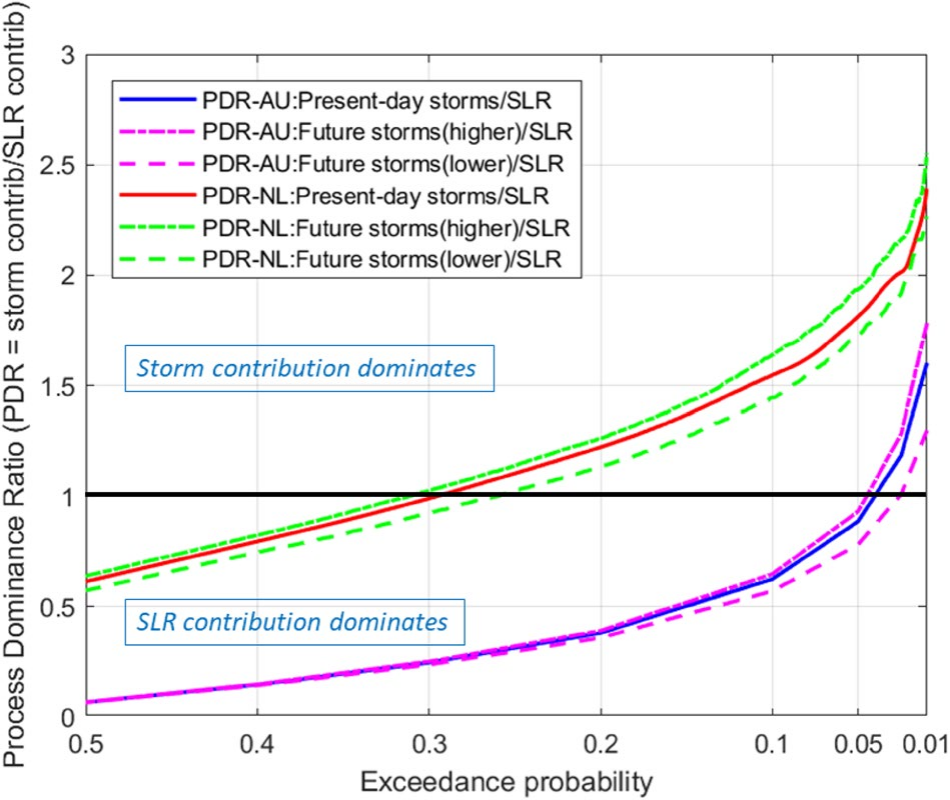
Process Dominance Ratio (PDR) versus exceedance probability illustrating the relative dominance of the contributions from sea level rise and storm erosion to total coastline recession by the end of the twenty-first century at Narrabeen beach, Sydney, Australia (annotated AU) and Noordwijk aan Zee strand, The Netherlands (annotated NL). PDR values above 1 indicate storm dominance and PDR values below 1 indicate SLR dominance.

Here, we introduce the Process Dominance Ratio (PDR), defined as:

1 $$PDR=\frac{Storm \; contribution \; to \; total \; recession}{SLR \; contribution \; to \; total \; recession},$$

which compares the relative contributions from storm erosion and SLR to total recession. A PDR value of greater than 1 indicates storm dominance while a PDR value less than 1 indicates the dominance of SLR on total recession. Figure 2 shows the Process Dominance Ratio (PDR) for the two sites considered here.

### Process dominance and implications

Inspection of Fig. 2 indicates that the question of whether SLR or storm erosion dominates twenty-first century coastline recession does not have simple answer. Rather, the answer depends on both the coastal type and the exceedance probability of interest (representing the risk tolerance level of decision makers). Figure 2 shows that SLR can be considered to dominate the amount of total recession at both coastal types when making moderately risk-averse decisions which can be based on the expected value of recession (i.e. 0.5 exceedance probability). Such decisions could for example be related to the placement of temporary coastal structures (e.g. summer beach cabins/restaurants) or low-cost recreational facilities. At the other extreme of very highly risk-averse decisions (e.g. construction of multi-storey apartments and tourist hotels, coastal infrastructure placement) that would typically accommodate the possibility of larger recessions with much lower exceedance probabilities, storm erosion can be considered to dominate over the SLR effect at both coastal types. Figure 2 shows that, while storm erosion starts to dominate over the SLR effect only for recessions with exceedance probabilities lower than about 0.05 at the swell dominated beach, the transition from SLR dominance to storm dominance occurs at a recession with much higher exceedance probability (0.3) at the storm dominated beach.

These results also have implications in terms of the modelling effort required to support different management decisions. For example, at both coastal types, management decisions that are willing to accept a 0.5 probability of an investment being subjected to erosion damage by 2100 may well be sufficiently supported by modelling SLR driven coastline recession in detail. However, a decision at a storm dominated beach concerning an investment that cannot accept a 0.5 probability of erosion damage (by 2100), careful consideration of storm erosion (in addition to SLR effects) is crucial in assessing total coastline recession. On the other hand, at a swell dominated beach, detailed consideration of storm erosion may become crucial only where decisions need to be extremely risk-averse and can only accept a very small probability (e.g. less than 0.05 by 2100) of erosion damage.

## Methods

### Study sites

#### Narrabeen beach, Sydney, Australia

**Figure 3:**
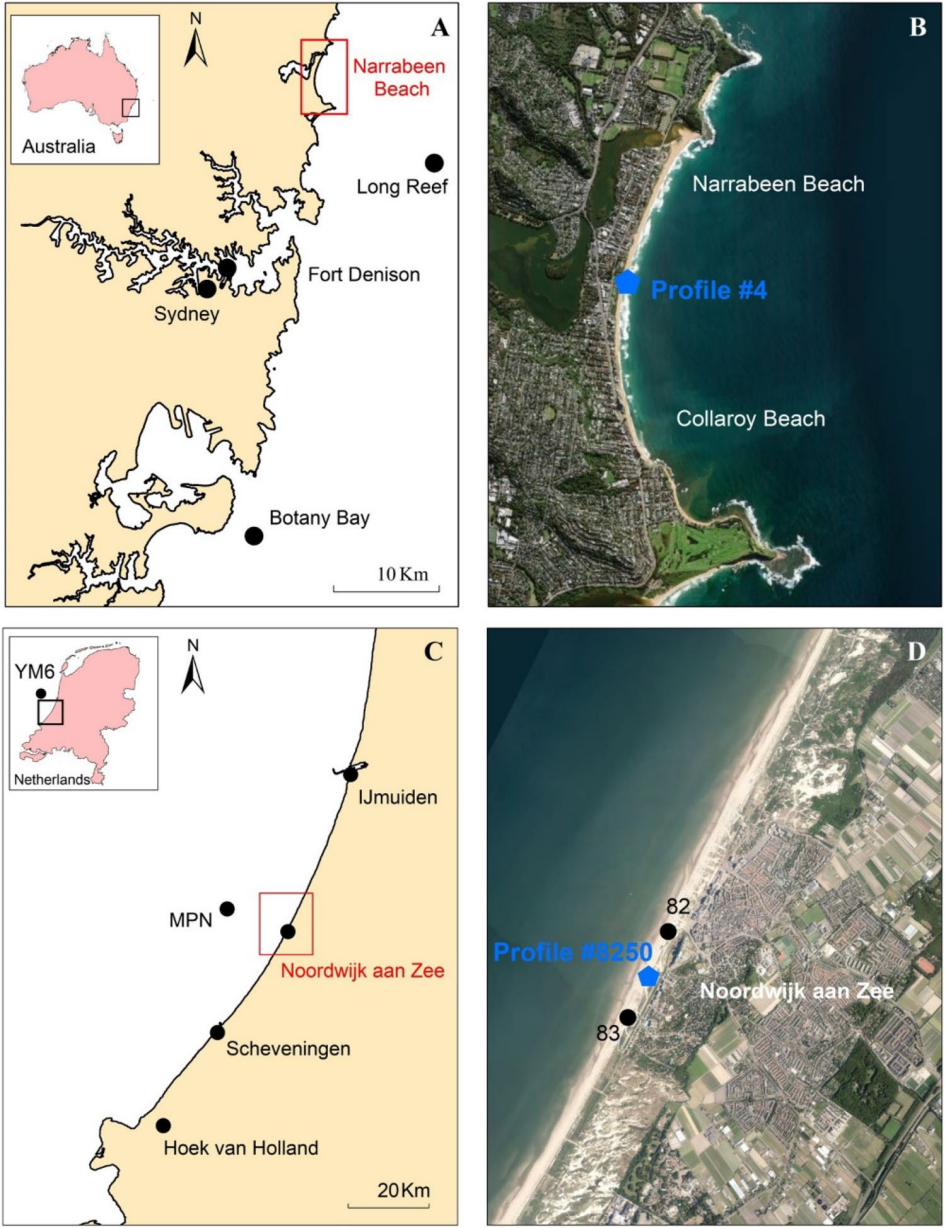
Study sites: (**a**,**b**) Narrabeen beach, Sydney, Australia (maps created with ArcGIS version 10.7.0.10348), (**c**,**d**) Noordwijk aan Zee strand, Netherlands (images from Google Earth).

Located about 20 km north of Sydney, the Narrabeen—Collaroy embayment comprises a 3.6 km sandy system bounded by Narrabeen headland to the North and Long Reef Point headland to the south (Fig. 3a,b). The sandy beach is composed of quartz sand with a median grain diameter *D*_50_ ≈ 0.3 mm^27^ and is backed by dunes. Tides in the region are semi-diurnal and microtidal^28^ and storm surges in the area are small^23,29^. All year round, Narrabeen is exposed to swells propagating from the southern ocean^28^ and as a result the wave climate at Narrabeen is swell dominated. The average significant wave height (*H*_*s*_) in the area is 1.6 m, with a peak wave period of 10 s, and the dominant wave direction is from the south-south-east (SSE). Although storms are more common during winter, there is no great seasonal variation in the wave climate^29,30^. Water level data is available since 1914 from the nearby Fort Denison tide gauge, while continuous wave data is available since 1971 from Botany Bay (non-directional) and Long reef (directional) gauges. Beach profiles have been surveyed at Narrabeen beach at a monthly frequency since 1976^31^. The results presented in this study pertains to the central profile, known as Profile #4, shown in Fig. 3b.

#### Noordwijk aan Zee strand, The Netherlands

Noordwijk aan Zee strand is a sandy beach located along the ~ 120 km long central Dutch coast, commonly known as the “Holland coast’” (Fig. 3c,d). The beach is backed by dunes^17,32^, and the median grain diameter is 0.15–0.25 mm^33^. Tides in the region are semi-diurnal and microtidal^34^. Storm surges in the North Sea can be large, with the 100 year return period surge elevation approaching 3.5 m^22^. The wave climate in the region is storm (sea) dominated, with swell (from NW) being present only about 20% of the time. The average significant wave height and wave period are 1 m and 6 s respectively, but wave climate is highly seasonal with larger storm waves (H_s_ > 1.5 m) during winter^33,35^. Wave and water level data are available from 1979 to 1992 at IJmuijden Munitiestrortsplaats (YM6) and from 1993 at Noordwijk Meetpost (MPN). The beach profile at Noordwijk has been monitored continuously since 1965 at an annual frequency under the Dutch government’s JARKUS program^32^, and is known as JARKUS profile #8250, shown in Fig. 3d.

### The PCR model

The PCR model (for a full model description please see Ranasinghe et al.^14^) computes the magnitude of coastline recession due to the combined effect of SLR and storms (storm waves and storm surge) by tracking the horizontal displacement of a chosen coastline position indicator, here taken as the dune position (toe of the dune), over a long time period, typically ~ 100 years. The model calculates the long-term net coastline recession due to the effect of successive storms superimposed on a slowly increasing mean water level (MWL) (i.e. SLR). As an illustration, assume that a 1 in 10 year storm occurs at the present time when the dune is at *x* = 0 (horizontal axis) and the mean water level (MWL) is at 0 m (relative to present-day MSL). Now assume that the erosion associated with this storm results in 10 m of dune retreat. If the next 1 in 10 year storm occurs after 10 years, SLR over this 10 year period would have resulted in the elevation of the MWL by 10 × (SLR/year). However, due to the slow nature of post-storm dune recovery, it is very unlikely that the dune would have completely recovered to its original position in the 10 year period between the two 1 in 10 year storms (i.e. hysteresis effect). Due to this hysteresis effect, say the dune only advanced 5 m seawards from its eroded position during the 10 year period between the two storms. When the second 1 in 10 year storm occurs under this situation of elevated MWL and a dune that is already 5 m landward of its present day position, at least an additional 10 m of dune retreat can be reasonably expected due to the second storm. The net dune retreat over the 10 year period then would be 15 m (10 − 5 + 10). As the MWL keeps increasing due to SLR, this process will repeat many times, leading to a net retreat of the coastline. The PCR model simulates this physical process, using the below described workflow).

1. Generate a long (typically ~ 100 years) time series of storms using Callaghan et al.’s^29^ JPM synthetic storm generator (see below for a brief description of the JPM approach).
2. Using IPCC sea-level rise projections, estimate the sea-level rise, relative to a benchmark value (typically beginning of the twenty-first century), at the time that each storm in the synthetic time series occurs.
3. For each storm in the generated storm time series, estimate coastline recession due to the combined effect of the storm and SLR at the time of each storm, while allowing for profile recovery between storms, using a physics based storm erosion model that is appropriate for the selected coastline indicator (in this application, Larson et al.’s^36^ dune impact model was used for Narrabeen beach (see Ranasinghe et al.^14^), while a combination of DUNERULE^37^ and Xbeach^38^ was used for Noordwijk aan Zee strand (see Li et al.^17^); the erosion models are calibrated for the respective sites using measured historical storm erosion data—for more details please see Ranasinghe et al.^14^ and Li et al.^17^). The combined effect of SLR and storm erosion is simulated in the PCR model by running the storm erosion model for each storm with the SLR induced elevation of MWL at the time of that storm.
4. Track and store the position of the coastline indicator throughout the simulation period.
5. Subtract the initial position of the coastline indicator from its final computed position (averaged over the last 2–3 years of the simulation) to estimate coastline recession during the simulation period (~ 100 years).
6. Repeat 1–5 until computed low exceedance probability (e.g. 0.05) recessions converge (i.e. bootstrapping).

The JPM (Joint Probability Model), adopted in step 1 of the PCR workflow above to generate the synthetic storm time series is fully described in Callaghan et al.^29^. This approach fits marginal, dependency and conditional distributions to long time series of forcing parameters (i.e. storm wave height, storm duration, storm wave period, storm wave direction, storm spacing, and storm surge), which are then used within a Monte Carlo simulation to derive a time series of storms and their associated characteristics.

### Limitations

This study concentrated only on sandy coasts, and as such the potential applicability of the results presented here are likely limited to the ~ 31% of the ice-free global coastline that is sandy^39^. At other types of coasts (e.g. muddy coasts, cliffed coasts), the relative dominance characteristics of storm erosion versus sea level rise on total coastline recession might be different from what is reported here.

While the two study sites considered here can be taken to represent end-members of sandy coast types that are more or less diametrically opposite with respect to wave and surge climate (Narrabeen—swell dominated, negligible surge; Noordwijk—storm dominated, high surge) as well as nearshore morphodynamics (Narrabeen—swash dominated; Noordwijk—drift dominated), there are many other intermediate types of sandy coasts within the full spectrum of the wide range of sandy coasts present around the world. Furthermore, different geologic controls and shelf dynamics may be present at different sites around the world.

The PCR model applications here do not consider alongshore gradients in longshore sediment transport or fluvial sediment supply to coasts. While these omissions do not affect the results at the two study sites, at other sites such process may need to be included in the model as sediment sources or sinks. Post-storm profile recovery is an important process that needs to be represented in PCR model simulations. Being a reduced complexity model, by design, the PCR model does not account for the full range of coastal forcing conditions that can be experienced (e.g., tides, waves, winds, ocean currents etc.) over the simulation period, but operates by running sequences of individual storms (together with SLR), with simplified inter-storm profile recovery parameterizations. Thus, the model does not account for potentially important long-term beach and dune migration processes that may be occurring. Although observed post-storm profile recovery rates were available (and used in the PCR applications) for the two case study sites described in the present study, this may not be the case at most other locations. Dastgheib et al.^16,20^ present a “reverse-engineering” solution that could be used in such situations to derive an estimate of average profile recovery rate via a few exploratory PCR simulations combined with observed coastline position change rates. However, what would be ideal is to use a process based, yet simple formulation that could estimate post-storm profile recovery rate at any location based on easily available parameters. To the authors knowledge, such a formulation doesn’t exist yet.

Another limitation of the present version of the PCR model is its assumption that the profile shape remains unchanged while it moved landwards (during storms) and seawards (during inter-storm recovery periods). While this does not account for increased erosion when a storm occurs on an accreted profile and decreased erosion when storms occur in quick succession on an already eroded profile, the net effect of such storm-by-storm differences in erosion would likely be small over a long simulation period, especially considering that a typical PCR simulation performs a 100-year simulation about 1000 times within the model’s Montecarlo framework. In terms of profile adjustment to SLR, PCR model applications do account for the raising of the dune toe and MWL in response to SLR, although this is done in different ways for different applications, as appropriate for the erosion model used in the PCR application (e.g. in the Narrabeen beach PCR application, the term that represents the vertical elevation difference between the dune toe and the start of swash in the Larson et al. (2004) dune impact model is increased by the amount of SLR that occurs between storm (*i*) and storm (*i *− 1), when computing the dune erosion for storm (i)).

The PCR model results presented in this study are limited, by design, to the end of the twenty-first century projections under the high-emission SSP5-8.5 climate scenario. Consideration of such a high-end scenario enables a clear separation of different system response characteristics to forcing, as was the focus of this study (i.e. determination of when storm erosion dominated over sea level rise, and vice versa, in terms of total coastline recession). The dominance of one process over the other may not be as clear for lower emission scenarios. In contrast, the relative dominance of SLR over storm erosion in total coastline recession would become even more clear for high-end SLR projections, such as for example, the low likelihood high impact (LLHI) SLR storyline presented in IPCC AR6.

## Data Availability

The data supporting the calculation and conclusions presented in this manuscript will be made available by the corresponding author, without reservation, to any qualified researcher.
